# Development of PROTACs to address clinical limitations associated with BTK-targeted kinase inhibitors

**DOI:** 10.37349/etat.2020.00009

**Published:** 2020-06-29

**Authors:** Rachael Arthur, Beatriz Valle-Argos, Andrew J. Steele, Graham Packham

**Affiliations:** 1 Cancer Research UK Centre, Cancer Sciences, Faculty of Medicine, University of Southampton, SO16 6YD Southampton, UK; 2 Institute for Life Sciences, University of Southampton, University Road, Highfield Campus, SO17 1BJ, Southampton, UK

**Keywords:** Chronic lymphocytic leukemia, B-cell receptor, signaling, BTK, ibrutinib, proteolysis targeting chimera

## Abstract

Chronic lymphocytic leukemia is a common form of leukemia and is dependent on growth-promoting signaling via the B-cell receptor. The Bruton tyrosine kinase (BTK) is an important mediator of B-cell receptor signaling and the irreversible BTK inhibitor ibrutinib can trigger dramatic clinical responses in treated patients. However, emergence of resistance and toxicity are major limitations which lead to treatment discontinuation. There remains, therefore, a clear need for new therapeutic options. In this review, we discuss recent progress in the development of BTK-targeted proteolysis targeting chimeras (PROTACs) describing how such agents may provide advantages over ibrutinib and highlighting features of PROTACs that are important for the development of effective BTK degrading agents. Overall, PROTACs appear to be an exciting new approach to target BTK. However, development is at a very early stage and considerable progress is required to refine these agents and optimize their drug-like properties before progression to clinical testing.

## Introduction

Chronic lymphocytic leukemia (CLL) is characterized by the accumulation of mature, clonal B cells and is the most common form of leukemia in adults with an annual incidence of ~5/100, 000 in the United States [1]. Treatment has been revolutionized in recent years by the introduction into routine clinical practice of the irreversible Bruton tyrosine kinase (BTK) inhibitor ibrutinib which interferes with signal transduction downstream of key cell surface receptors, including the B-cell receptor (BCR), a major driver of this and other forms of B-cell neoplasia. However, despite the dramatic clinical responses that can be induced by ibrutinib, toxicity and resistance are major limitations and new treatment options are required.

Proteolysis targeting chimeras (PROTACs) are an exciting new approach for targeted inhibition based on event-driven rather than occupancy-based activity [2, 3]. Several BTK-targeted PROTACs have been described and these studies provide a powerful illustration of how PROTACs can address clinically relevant drawbacks associated with ibrutinib, including improved selectivity and retained activity in models of ibrutinib resistance. In this review, we provide an introduction to CLL, the role of BTK in signaling downstream of the BCR and its inhibition by ibrutinib, including its clinical activity. We then describe recent findings with BTK-targeted PROTACs focusing on how these studies reveal principles underlying efficient PROTAC design and how current agents may provide improvements over ibrutinib.

## An introduction to PROTACs

This section provides a brief introduction to PROTAC technology. Readers are referred to other expert reviews in this special issue for further details.

**Figure 1:**
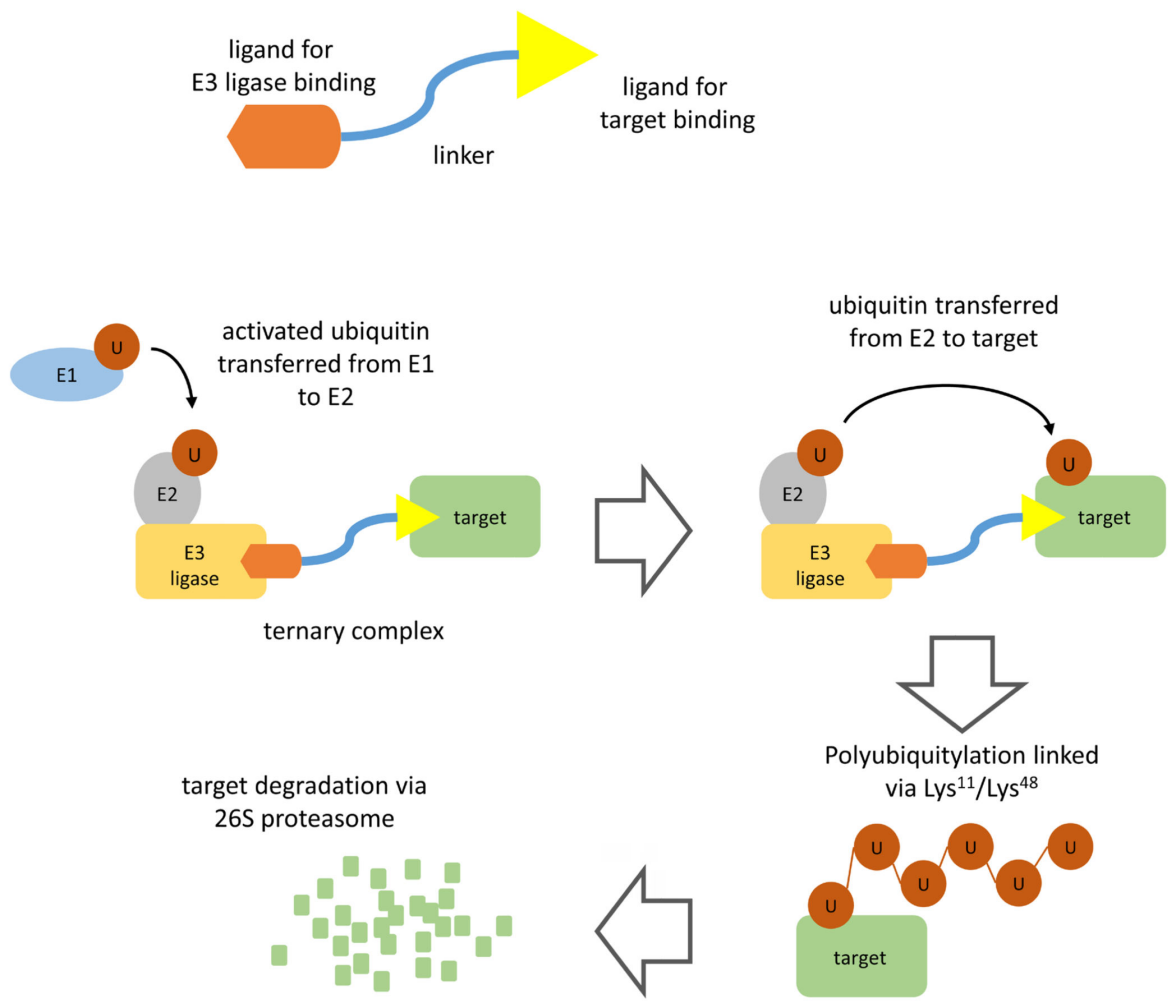
PROTAC-mediated target degradation. The figure illustrates the basic structure of a PROTAC (top) and the mechanism of PROTAC-mediated target degradation (bottom). Interaction of the PROTAC with its target and an E3 ligase (TC formation) facilitates multiple rounds of target ubiquitylation followed by target degradation via the 26S proteasome. The PROTAC and E3 ligase are released and are therefore available for further rounds of PROTAC-mediated degradation

PROTACs comprise a warhead directed against the target of interest (in this case BTK) coupled through a variable linker to an ubiquitin protein ligase (E3) ligase-recruiting element (Figure 1). This facilitates formation of a ternary complex (TC) comprising the target, PROTAC and an E3 and results in degradation of the target via the proteasome.

Polyubiquitylation of the target protein is catalyzed by a family of enzymes termed ubiquitin-activating enzyme (E1), ubiquitin-conjugating enzyme (E2) and E3 [4]. Ubiquitin is first activated by E1 biquitin-activating enzyme which catalyzes formation of a thioester linkage between the C-terminus of ubiquitin and a cysteine residue within the E1 protein. Activated E1-bound ubiquitin is then transferred to an E2. Finally, activated ubiquitin is transferred from E2 to a lysine residue within the target protein via the action of an E3 ubiquitin ligase. There are > 500 E3 ligases which differ in their ability to recognize specific degradation signals within target proteins. E3 ligases commonly targeted by PROTACs include cereblon (CRBN), murine double-minute 2 (MDM2), Von Hippel-Landau (VHL), and inhibitor of apoptosis protein (IAP) [2, 3]. Following transfer of ubiquitin to the target protein, E1 continues to recruit and activate further ubiquitin molecules allowing E2 to form a chain of ubiquitin molecules on the target protein whereby the C-terminus of each ubiquitin is linked to a lysine residue (either Lys11 or Lys48) within the preceding ubiquitin molecule. It is this Lys11/Lys48-linked polyubiquitin chain which serves as a target for recognition by the proteasome.

The key distinction to conventional inhibitors is that PROTACs operate via “event-driven” pharmacology [2, 5]. Thus, transient target: PROTAC binding is sufficient to recruit an E3 ligase resulting in a biological effect. Moreover, subsequent release of PROTAC means that these agents can act as catalysts to inhibit target function. By contrast, conventional inhibitors act stoichiometrically via “occupancy-driven” pharmacology.

Although firm “rules” for development of effective PROTACs are lacking it is clear that activity is not simply determined by the binary affinities of the PROTAC for its target or E3-ligase. Selectivity can be influenced by additional stabilizing protein-protein interactions within the TC which can enhance relative degradation of targets with weak affinity for the PROTAC [6–9]. On the other hand, PROTACs may be inactive despite formation of the TC if this complex does not allow transfer of ubiquitin to an appropriately-positioned lysine residue within the target protein. Thus, choice of warhead, linker and E3 ligase-recruiting ligand all exert an important influence and require optimization. Importantly, these elements are coupled, so the choice of optimal linker will depend on the nature of the coupled warhead/E3 ligase-recruiting ligand.

Due to their multi-domain nature, PROTACs are relatively large and development of agents with physiochemical properties suitable for clinical administration is likely to be challenging [10]. For example, even the most effective PROTACs typically have greater molecular weight, total polar surface area and numbers of hydrogen bond acceptors and rotatable bonds compared to classical, orally available small molecule drugs [11]. These drawbacks may be particularly problematic for kinase-targeting PROTACs where comparator small molecule kinase inhibitors (including ibrutinib) are typically administered orally. Although some physiochemical limitations may be offset by the unique pharmacological activities of PROTACs (for example, reduced target occupancy due to poor cell permeability may be less problematic due to their catalytic mode of action), studies have shown that it is possible to substantially improve the activity of PROTACs through targeted medicinal chemistry efforts [12].

## CLL

CLL is a common, low-grade B-cell malignancy characterized by the accumulation of mature B cells in the blood, bone marrow and secondary lymphoid organs of patients [1]. The clinical course is highly heterogeneous with some patients not requiring immediate treatment and managed by “watchful waiting”. Progressive disease typically follows a response/relapse course whereby patients require repeated rounds of treatment which are followed by relapse and ultimately succumb to the disease.

The disease can be divided into two major subgroups depending on whether the B-cell-of-origin from which the leukemia derives has undergone somatic hypermutation (SHM). SHM is a natural process in normal B cells whereby mutations are introduced into the genes encoding the variable regions of the BCR, the antigen receptor for B cells, and is important for generation of high affinity antibodies. Importantly, these CLL subgroups have very different outcomes and 10-year survival rates are ~30-35% for cases lacking SHM [termed unmutated CLL (U-CLL)] and ~85% for cases with SHM [termed mutated CLL (M-CLL)] [1].

The accumulation of malignant cells in CLL appears to be driven by both intrinsic genetic alterations and a tumor-promoting effect of the microenvironment [1, 13]. The most common chromosomal changes include deletion of part of chromosome 13q (resulting in overexpression of the anti-apoptotic BCL2 protein due to loss of repressing miRNAs within this chromosomal region), deletions of 17p or 11q (which result in defective DNA damage responses due to loss of genes encoding p53 or ATM) or trisomy 12 (the functional consequences of which are not well understood) [14–16]. In addition, next generation sequencing has identified recurrent somatic mutations, the most common of which are loss-of-function mutations of p53 or ATM, activating mutations of *NOTCH1* (which leads to enhanced NOTCH1 signaling) and mutations of *SF3B1* (which encodes a splicing factor) and *XPO1* (which encodes a protein involved in nuclear export of proteins and RNAs) [17, 18]. Except for del(13q), these chromosomal alterations and somatic mutations are more common in the more aggressive U-CLL disease subgroup. The mutational burden also increases with treatment due to selection of drug resistant subclones. However, the overall burden of somatic mutations in CLL is relatively low compared to other B-cell malignancies.

In addition to genomic drivers, the tissue microenvironment appears to play a central role in driving accumulation of malignant cells. CLL cell proliferation occurs within “proliferation centers”, microanatomical sites within secondary lymphoid organs where CLL cells interact with an array of supporting cells, including stromal cells, nurse-like cells (a type of macrophage) and T cells [19]. These promote CLL cell proliferation and survival via a range of factors, including CD40L, CXCL12, IL4, BAFF and contact with integrins. Cell communication within tissues is bi-directional and CLL cells also secrete factors which influence the microenvironment, such as the T-cell attractants CCL3 and CCL4. Tissues are also the main site of antigen engagement of the BCR of CLL cells (discussed in detail below) [20]. Importantly, there is exchange of CLL cells between the blood and tissues which is mediated, as in normal B cells, by an array of chemokine receptors and integrins [21, 22].

Treatment for CLL has evolved rapidly but once progression occurs the main treatment remains chemoimmunotherapy (CIT) which is typically based on an anti-CD20 antibody (rituximab, ofatumumab or obinutuzumab) combined with either fludarabine or chlorambucil for less fit patients [1]. Many patients have a good initial response to CIT but ultimately relapse and require further rounds of treatment. Moreover, patients with *p53/ATM* mutations do not respond to CIT and alternative therapeutic strategies are required. In these cases, patients may be treated with newer targeted agents, including BCL2 inhibitors such as venetoclax to reverse BCL2-mediated suppression of apoptosis [23] or inhibitors directed against BCR-associated signaling kinases, including ibrutinib (discussed in detail below) [24].

## The BCR in CLL

**Figure 2:**
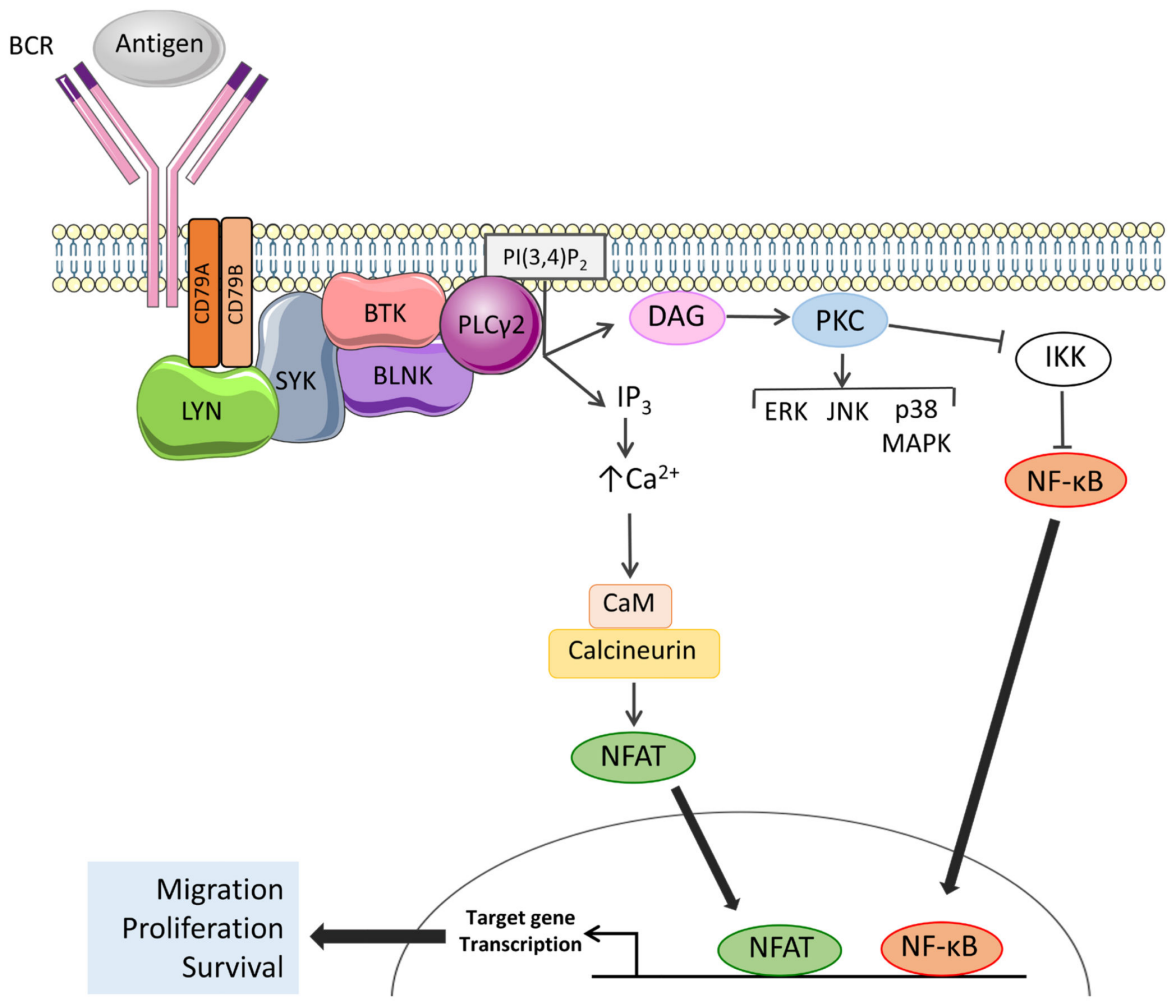
Activation of BTK and PLCγ2 downstream of the BCR. The BCR comprises an antigen-binding immunoglobulin coupled to CD79A and CD79B signal transduction molecules. BCR engagement leads to activation of proximal kinases, such as LYN and SYK. BTK is activated by SYK-mediated phosphorylation and autophosphorylation and, with the scaffold protein BLNK, mediates activation of PLCγ2. Once activated, PLCγ2 cleaves phosphatidylinositol 4,5-bisphosphate (PI(4,5)P2) to generate inositol 1,4,5-trisphosphate (IP_3_) and diacylglycerol (DAG), leading to increased intracellular Ca^2+^ (iCa^2+^) and activation of PKC isoforms. Key downstream effects include activation of MAP kinases and Ca^2+^-dependent transcription factors, such as nuclear factor-κB (NF-κB) and, via calmodulin (CaM) and calcineurin, nuclear factor of activated T-cells (NFAT), resulting in increased transcription of genes involved in control of survival, migration and proliferation. Note that not all pathways activated downstream of the BCR are shown

The BCR is the key functional determinant of normal B cells and continues to play a major role in determining the behavior of CLL cells post-transformation [25]. The BCR consists of a surface immunoglobulin which confers antigen-binding specificity coupled to two transmembrane signal transduction molecules, CD79A and CD79B (Figure 2). Antigen engagement results in phosphorylation of conserved tyrosine residues within the cytoplasmic domains of CD79A and CD79B by the kinase LYN, leading to recruitment and activation of a second kinase, SYK. This is followed by assembly of a multiprotein complex termed the signalosome resulting in activation of protein and lipid kinases (e.g., BTK and PI3K), adaptor proteins (BLNK) and other enzymes [e.g., phospholipase C (PLCγ2)] [26].

The BCR “senses” the environment for molecules that bind with significant avidity and the strength and nature of the response following receptor engagement is varied [27]. Depending on the degree of stimulation and the context in which engagement occurs, antigen binding to normal B cells can trigger signaling responses leading to survival/proliferation, apoptosis or anergy, a state of cellular lethargy induced by antigen engagement in the absence of T-cell help [27]. BCR also mediates a low-level “tonic” signal which is essential for B-cell survival. In CLL, two differing fates appear to play a central role in determining the heterogeneous clinical course. Thus, BCR-induced anergy predominates in M-CLL whereas antigen-induced pro-proliferative/survival signaling is more apparent in U-CLL and may mediate, at least in part, the more progressive nature of this subgroup [28].

## BTK

BTK is a TEC family non-receptor kinase that plays a critical role in B-cell development. Individuals with X-linked agammaglobulinemia (XLA) lack BTK expression due to loss-of-function germline mutations and this leads to absence of blood B cells and low levels of serum immunoglobulins. BTK plays a pivotal role in mediating signal transduction downstream of the BCR where it is activated following phosphorylation on Tyr^551^ by LYN and/or SYK (Figure 2) [29, 30]. In some settings, BTK activation also involves PI3K activity which leads to accumulation of the inositol lipid phosphatidylinositol 3,4,5-trisphosphate (PI(3,4,5)P_3_) and recruitment of BTK to the plasma membrane via its PI(3,4,5)P_3_-binding PH domain [31]. BTK autophosphorylation on Tyr^223^ results in full activation [29].

The principle downstream target for BTK is PLCγ2 which catalyzes conversion of (PI(4,5)P_2_) to DAG and IP_3_ (Figure 2). PLCγ2 activation is mediated by BTK-dependent phosphorylation and is facilitated by BLNK which provides a scaffold for BTK/PLCγ2 interactions [32–34]. Production of IP_3_ downstream of BTK/PLCγ2 results in increased intracellular Ca^2+^ (iCa^2+^) by the release of Ca^2+^ from the endoplasmic reticulum via IP_3_ receptors followed by influx of extracellular Ca^2+^ via plasma membrane channels in a process known as store-operated Ca^2+^ entry [35]. Increased iCa^2+^ together with DAG accumulation leads to activation of protein kinase C isoforms which mediate activation of various MAPKs (ERK, JNK and p38 MAPK), and nuclear translocation of the transcription factor NF-κB (by inhibition of its inhibitor IKK) [36, 37]. Increased iCa^2+^ also activates CaM and calcineurin leading to nuclear translocation of NFAT. NF-κB and NFAT in turn induce expression of target genes encoding proteins which promote B-cell survival (*BCL2*, *BCL2A1*), migration (*CCR7*) and proliferation (*MYC*, *CCND1* and *CCND2*) [38–41]. BTK also mediates receptor “cross-talk” downstream of the BCR and contributes to activation of integrins and increased cell adhesion [42] and down-regulation of chemokine receptor (CXCR4) following BCR activation [43].

It is important to recognize that BTK also contributes to signaling downstream of other cell surface receptors in B cells, including chemokine and toll-like receptors [44–47]. For example, deletion or chemical inhibition of BTK reduces CXCL12 (the major ligand for CXCR4)-mediated cell migration and adhesion, and homing of B cells to lymphoid organs *in vivo*. BTK also has important functions in non-B cells. For example, BTK is important for FcγR-induced activation of pro-inflammatory cytokines in monocytes [48] and FcγRIIA-induced platelet activation [49].

## Ibrutinib

**Figure 3:**
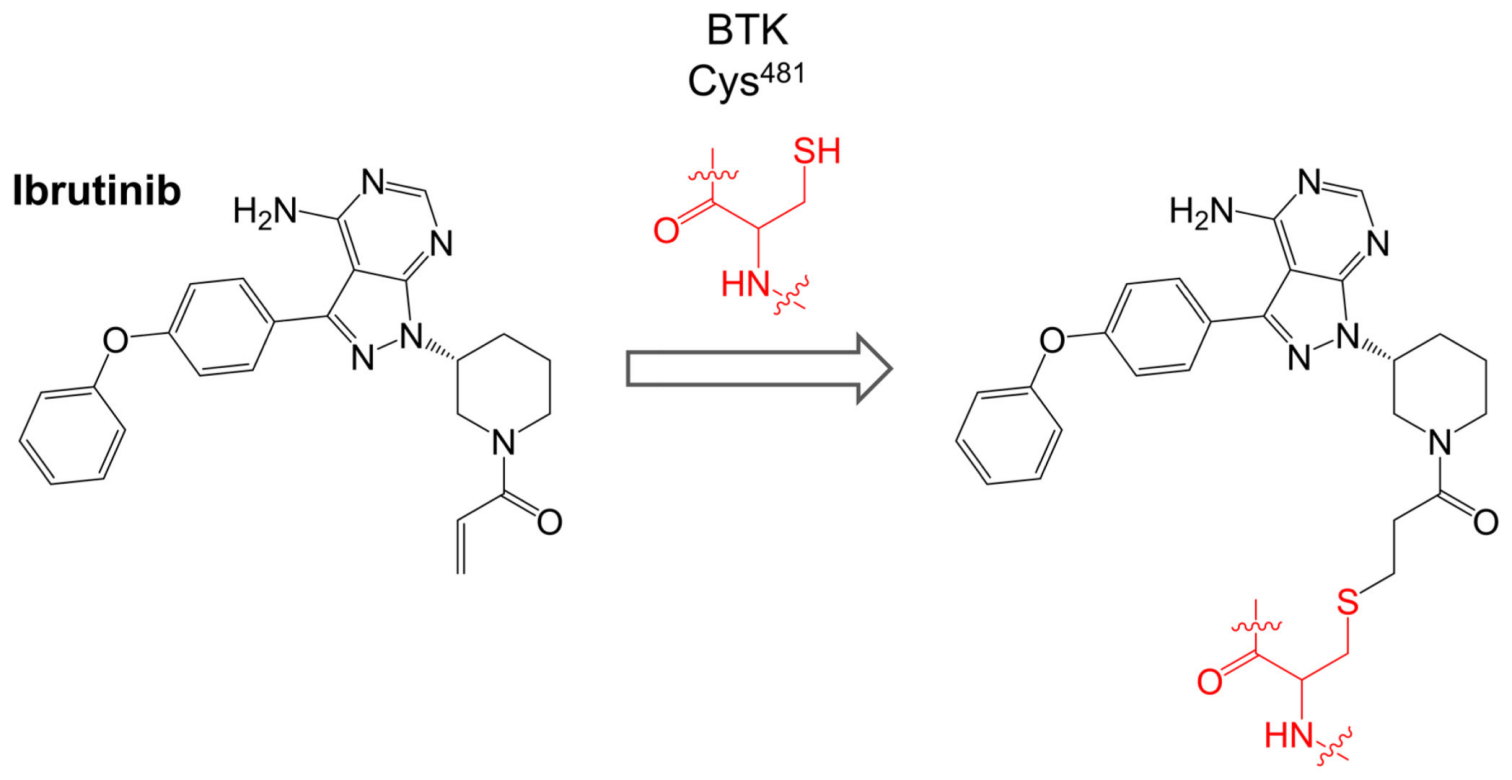
Reaction of ibrutinib with Cys^481^ of BTK

Ibrutinib (previously known as PCI-32765) is a first-in-class oral, once daily inhibitor of BTK. It binds covalently to Cys^481^ within the BTK active site via reaction between the cysteine thiol and the ibrutinib acrylamide group (Figure 3). It is a potent inhibitor of BTK (50% inhibition at ~0.5 nM in an *in vitro* kinase assay) [50].

Ibrutinib inhibits many other kinases in addition to BTK including BLK and BMX (which are inhibited with similar potency to BTK, IC_50_ < 1 nM) and HCK, EGFR, ERBB2, ITK and JAK3 (IC_50_ 1-20 nM) [50]. Of these BLK, BMX, EGFR, ERBB2, ITK and JAK3 contain cysteine residues analogous to Cys^481^ of BTK. In initial cell-based studies, ibrutinib inhibited BTK autophosphorylation on Tyr^223^ in anti-IgM-stimulated DOHH2 human lymphoma cells with an IC_50_ of ~10 nM [50]. Ibrutinib also inhibited downstream phosphorylation of PLCγ2 and ERK1/2.

The first characterization of ibrutinib’s effects in primary CLL cells was reported by Herman et al. [51]. This study confirmed that ibrutinib reduced BTK tyrosine phosphorylation and downstream phosphorylation of AKT and ERK1/2, and activation of NF-κB. Although ibrutinib’s effects on CLL cell viability were generally modest (~10% median cell killing at 1 µM), ibrutinib induced caspase-dependent apoptosis in both U-CLL and M-CLL cases, and independently of the presence of del(13q), del(11q) or del(17p). Ibrutinib also induced apoptosis in the presence of various survival signals, including CD40L, BAFF, fibronectin or co-culture with stromal cells. Importantly, this study showed that ibrutinib reduced production of cytokines (IL6, IL10, and TNFα) from activated T cells, despite an apparent lack of BTK expression in these cells, revealing potential for off-target effects.

Ponader et al. [52], demonstrated that ibrutinib inhibited CLL cell survival induced by either anti-IgM (to cross-link and stimulate the BCR) or co-culture with nurse-like cells. Ibrutinib also reduced CLL cell proliferation and secretion of CCL3/4 following nurse-like cell co-culture. Importantly, CCL3/4 concentrations were also reduced in the plasma of patients receiving ibrutinib suggesting that CCL3/4 could be used as a pharmacodynamic (PD) response biomarker. Consistent with previous studies which revealed a role for BTK in mediating signal transduction downstream of chemokine receptors in normal B cells [44, 45], ibrutinib inhibited migration of CLL cells towards CXCL12/CXCL13 and inhibited AKT/ERK phosphorylation following stimulation with anti-IgM, CXCL12 or CXCL13. Finally, this study showed that ibrutinib reduced the accumulation of leukemic cells *in vivo* in the Eµ-TCL1 mouse model of CLL [52].

De Rooij et al. [53], demonstrated that ibrutinib inhibited BCR-induced PLCγ2 phosphorylation, integrin-mediated adhesion to fibronectin or VCAM-1 and CXCL12/CXCL13/CCL19-induced adhesion, and migration.

Finally, ibrutinib has been shown to interfere with downstream signaling responses, including anti-IgM-induced pro-survival survival signaling, and induction of mRNA translation and a cytoprotective unfolded protein response [54–56]. Interestingly, potentiation of BCR signaling by IL4 was shown to render cells less sensitive to pro-apoptotic effects of ibrutinib [57].

It is important to consider off-target effects of ibrutinib. This is of course relevant for drug toxicity and this is discussed in detail below. However, off-target effects might also be beneficial and contribute to ibrutinib’s efficacy in CLL or broaden the drug’s therapeutic utility. For example, inhibition of ITK in T cells appears to promote beneficial Th1 immunity, and both BTK-dependent and -independent effects appear to contribute to enhanced T cell numbers and function in CLL patients [58, 59]. Moreover, ibrutinib has activity in models of solid tumors, for example, by inhibition of EGFR/HER2 in EGFR mutant non-small cell lung cancer [60], and is also undergoing clinical evaluation in non-hematological malignancies.

## Clinical trial data in CLL

Initial clinical evaluation of ibrutinib was carried out in a phase I trial of patients with relapsed/refractory (R/R) B-cell malignancies which included 16 patients with CLL or small lymphocytic lymphoma (SLL, a tissue-based variant of CLL) [61]. The study reported a striking response rate amongst CLL/SLL patients for this heavily pre-treated population; 11/16 (69%) achieved objective responses (complete or partial response) assessed using standard response criteria. Notably, all responding CLL patients experienced a rapid reduction in their lymph node size and disease burden accompanied by lymphocytosis (i.e. increased CLL cells in the blood) lasting over a year in some patients. This effect is thought to be due to redistribution of cells from tissues to blood rather than a sign of disease progression and has been reported in other clinical trials of signal inhibitors in CLL patients and in studies of ibrutinib in mouse models [52]. It is likely to be due to effects of ibrutinib on the pathways that control homing/retention of CLL cells within tissues, potentially via effects on chemokine receptors and integrins. This lymphocytosis complicates assessment of response and subsequent trials have adopted modified assessment criteria.

Another notable feature reported in this first clinical study of ibrutinib was that BTK occupancy by drug was maintained at high levels (> 80%) for 24 h, whereas plasma concentrations of ibrutinib rapidly decreased following drug administration [61]. This is likely to be explained by the covalent nature of the interaction between ibrutinib and BTK. Based on these very promising clinical responses observed in this trial, more advanced trials were performed (summarized in Table 1) and these ultimately led to the approval of ibrutinib in the United States, Europe and other countries for treatment of treatment naïve (TN) and R/R CLL.

## Limitations of ibrutinib

Although ibrutinib has led to major improvements in outcomes for CLL patients, substantial limitations have been encountered, especially linked to development of resistance and toxicity. Rates of discontinuation vary between clinical settings but may be as high as ~50% at 3 years [71]. Outcomes following ibrutinib discontinuation are poor, especially for patients who discontinue due to progression, but this may improve with increasing availability of other further treatment options (e.g., BCL2 inhibitors) [72, 73].

Resistance is either simple (i.e. progression of CLL) or transformation to higher grade malignancy. Approximately 80% of progressive cases have mutations of either BTK or PLCγ2 whereas the frequency of these mutations is lower (~40%) in cases that transform. The vast majority of BTK mutations involve substitution of Cys^481^ with a serine residue. Cys^481^Ser mutant BTK retains enzymatic activity but is much less sensitive to ibrutinib-mediated inhibition due to the lack of covalent binding [74]. It remains to be definitively demonstrated that these mutations cause clinical ibrutinib resistance [75] but it is likely that their acquisition represents progression of disease to a state that retains BTK-dependence but reduced sensitivity to (reversible) ibrutinib-mediated inhibition. PLCγ2 mutations affect a number of different amino-acid residues but are thought to maintain activity despite BTK inhibition [76].

The most common side effects of ibrutinib include diarrhea, nausea, fatigue, upper respiratory tract infections, rash, dyspnea, and edema. More severe toxicities which lead to discontinuation include arthralgia, atrial fibrillation and rash in the front-line setting, and atrial fibrillation, infection, pneumonitis, bleeding and neutropenia in R/R disease [71]. These toxicities may be mediated by off-target effects. For example, off-target EGFR inhibition by ibrutinib is thought to contribute to development of rash and diarrhea. Atrial fibrillation is a serious complication and may have an incidence of up to 10% in ibrutinib-treated patients at 2 years follow-up [77]. The mechanisms are not known, but cardiac tissue does express BTK (as well as TEC which is also effectively inhibited by ibrutinib) [78]. Bleeding is another common cause for discontinuation. Interestingly, XLA patients do not have bleeding issues [79] implying factors other than BTK contribute to this toxicity. This may involve TEC which, like BTK, is involved in collagen receptor glycoprotein VI signaling and platelet aggregation [80, 81]. Consistent with this, incidence of bleeding is less with acalabrutinib, a more selective, second generation irreversible BTK inhibitor.

Overall, ibrutinib is a powerful new therapeutic agent for CLL. It can induce dramatic clinical responses, however, resistance and toxicity are substantial limitations. There are numerous new BTK inhibitors in development, including acalabrutinib and non-covalent inhibitors, such as vecabrutinib and AQR-531 [82]. However, PROTACs offer an exciting new approach to targeted inhibition of BTK and to address clinical limitations associated with ibrutinib.

## BTK-targeted PROTACs

**Figure 4:**
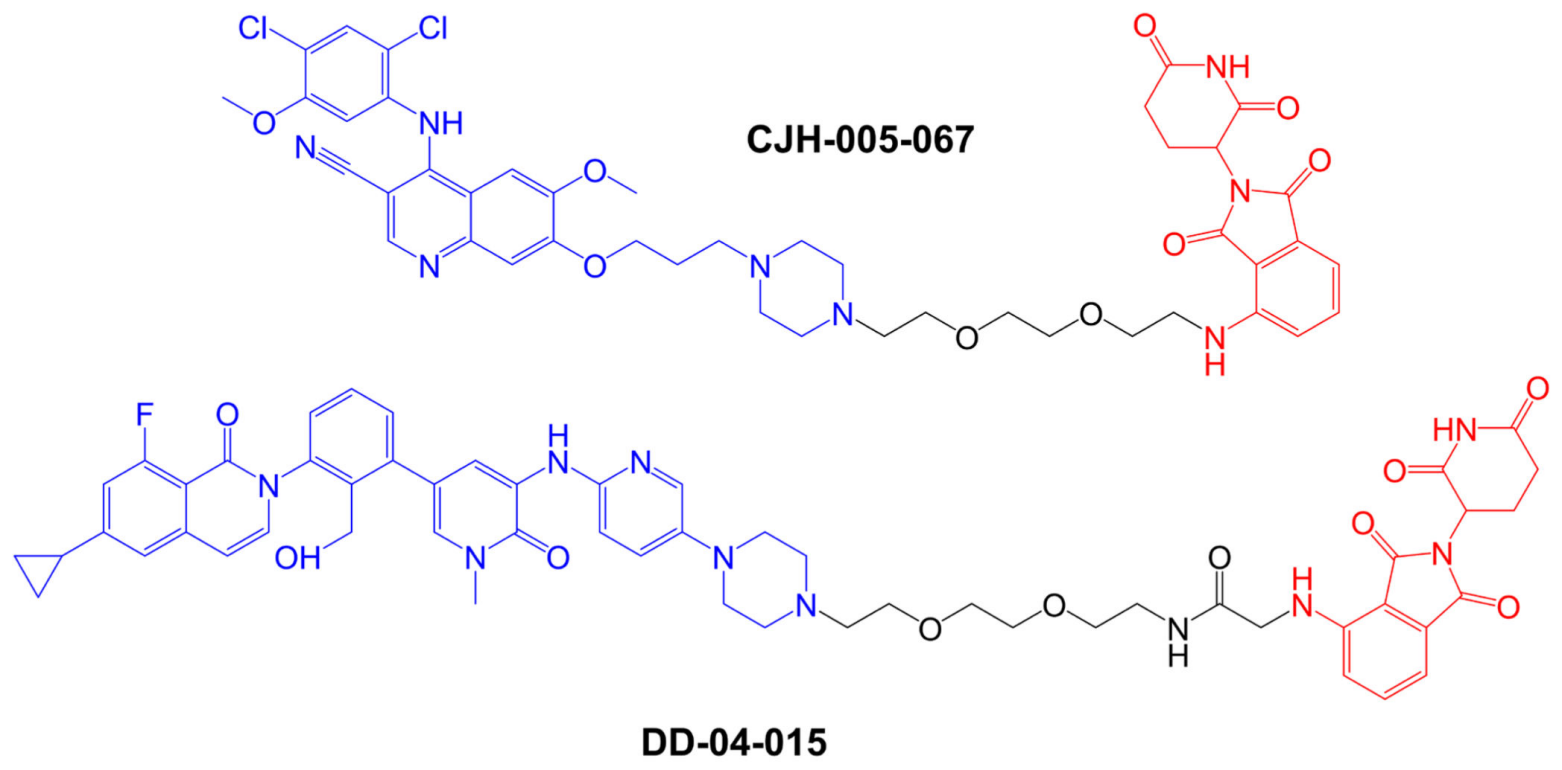
Structures of compounds CJH-005-067 and DD-04-015. BTK-targeted warheads (derived from bosutinib in CJH-005-067 and RN486 in DD-04015) are colored blue and pomalidomide-derived CRBN-targeting moieties are colored red [8]

The first specifically designed BTK-targeted PROTACs were reported by Huang et al. [8], at the start of 2018. CJH-005-067 and DD-04-015 contain warheads derived from the non-covalent BTK inhibitors bosutinib (BCR-ABL kinase inhibitor known to also inhibit BTK [83]) and RN486 (a highly selective BTK inhibitor [84]), respectively, and a shared pomalidomide-derived E3 ligase-binding moiety (Figure 4). Pomalidomide recruits CRBN, the substrate recognition component of the DCX E3 protein ligase complex [85].

Both compounds very effectively reduced expression of BTK within 4 h in acute myeloid leukemia (AML)-derived MOLM-14 cells with little effect on other potential PROTAC targets investigated, including AURKA. DD-04-015 was most effective at ~100 nM but showed a pronounced “hook effect” where the effectiveness of BTK down-modulation was reduced at higher concentrations (10 µM). A hook effect is thought to be due to predominant formation of dimer target:PROTAC and PROTAC:E3 ligase complexes which fail to engage target proteolysis, rather than TC formation, at high PROTAC concentrations.

When tested for growth inhibitory activity in BTK-dependent TMD8 cells (derived from diffuse large B-cell lymphoma), DD-04-015 and its parental BTK inhibitor (RN486) showed similar potency (IC_50_s ~20-30 nM following a 3 day exposure). However, the potency of DD-04-015 was relatively effectively maintained following wash-out of drug after a relatively short (6 h) exposure compared to RN486. This prolonged PD effect of DD-04-015 relative to RN486 was ascribed to persistent effects following BTK degradation compared to reversible active site occupancy. Similar prolonged PD effects have been described for PROTACs targeted against other targets [86].

Buhimschi et al. [87], described a series of BTK-targeted PROTACs utilizing a non-covalent BTK inhibitor warhead which was closely related to ibrutinib but lacked the acrylamide moiety which mediates covalent binding of ibrutinib to BTK Cys^481^. This inhibitor was coupled to various linkers and a pomalidomide-derived CRBN-targeting motif. The initial compounds, MT-540 and MT-541, had 12-atom linkers and very effectively reduced BTK expression in Namalwa cells (derived from Burkitt’s lymphoma). Exploration of the effect of shortening the linker demonstrated that an 11-atom linker was most effective for BTK targeting with a DC_50_ (i.e. concentration required to degrade 50% of the total BTK pool) of ~70 nM. However, more drastic reductions in linker length (to 8 or 5 atoms) ablated PROTAC activity.

**Figure 5:**
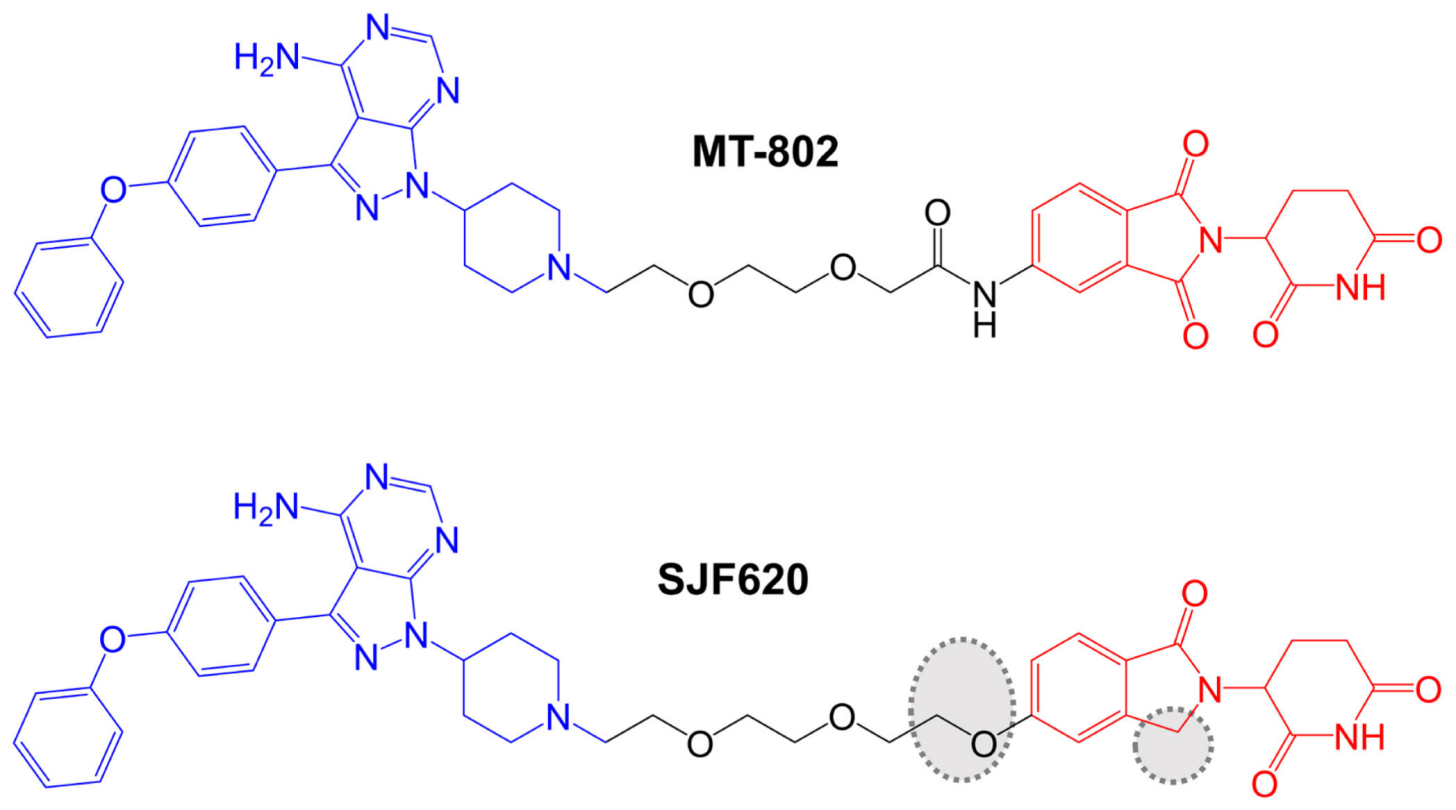
Structures of compounds MT-802 and SJF620. The reversible BTK-inhibiting warhead related to ibrutinib is colored blue and the CRBN-targeting moieties are colored red [87, 88]. Differences in the structure of SJF620 compared to MT-802 are highlighted by dotted regions

Further compounds were synthesized using a different point on the pomalidomide moiety for linker attachment (C5 of the phthalimide ring *versus* C4) (Figure 5). With this attachment position, inclusion of an 8-atom linker in compound MT-802 resulted in very potent activity (DC_50_ ~9 nM in a 24 h assay in Namalwa cells), whereas this linker length was incompatible with PROTAC activity in the context of C4-attachment. A 12-atom linker attached at C5 (compound MT-809) also resulted in potent activity (DC_50_ ~12 nM). MT-802 and MT-809 very effectively induced BTK degradation (> 99% maximal reduction at 250 nM) and did not show a hook effect at concentrations up to 2.5 µM. Interestingly, BTK-targeted PROTACs based on a VHL-binding ligand were relatively inactive for BTK degradation. Therefore, effective BTK targeting is dependent on selection of an appropriate E3 ligase binding moiety.

More detailed biological characterization focused on MT-802 and a related control compound SJF-6625 which is unable to bind CRBN due to methylation of the glutarimide ring of the pomalidomide moiety and does not induce BTK degradation. Time course experiments demonstrated that MT-802-induced BTK degradation was rapid (detected within 1 h and near maximal at 4 h with 250 nM MT-802). MT-802-induced BTK degradation was effectively prevented by the proteasome inhibitor epoxomicin or MLN-4924, an inhibitor of the NEDD8-activating enzyme that neddylates and activates the CRBN complex. Moreover, BTK degradation was prevented by an excess of either ibrutinib or pomalidomide confirming activity was dependent on formation of a TC.

Analysis of the specificity of MT-802 for kinase binding was performed using KINOMEscan (DiscoverX®), a competition based assay to measure binding of 468 kinases to an immobilized ligand. Compounds that bind the kinase active site (i.e. ibrutinib and MT-802) prevent binding of the kinase to immobilized ligand reducing the amount of kinase captured. The amount of kinase captured can be compared between test *versus* control samples, and quantitative polymerase chain reaction is used to detect the specific kinases (each tagged with a unique DNA label) bound to the immobilized ligand. This demonstrated that, like ibrutinib, MT-802 also bound TEC. However, a number of other ibrutinib-targeted kinases were only relatively weakly bound by MT-802, including ITK and JAK3. The increased target-specificity of MT-802 relative to ibrutinib was thought to be, at least in part, due to the non-covalent nature of the BTK-targeted warhead in MT-802.

The study by Buhimschi et al. [87], is particularly interesting as it included the first analysis of effects of BTK-targeted PROTACs on Cys^481^Ser mutant BTK, acquisition of which (as described above) is thought to be a major cause of ibrutinib resistance in CLL patients. *In vitro* kinase assays demonstrated that although MT-802 was a less potent inhibitor of wild-type BTK than ibrutinib (IC_50_s for MT-802 and ibrutinib were ~50 nM and < 0.05 nM, respectively), MT-802, but not ibrutinib, retained activity against Cys^481^Ser mutant BTK (IC_50_s for MT-802 and ibrutinib were ~20 nM and ~2 nM, respectively). Moreover, when overexpressed in BTK-null XLA cells, wild-type and Cys^481^Ser mutant BTK were approximately equally sensitive to MT-802-induced degradation (DC_50_s ~15 nM). Perhaps most importantly, MT-802 retained the ability to reduce the level of the active autophosphorylated form (i.e. BTK kinase activity) of BTK in patient-derived CLL cells from an ibrutinib-resistant patient with Cys^481^Ser mutated BTK. By contrast, ibrutinib was only able to reduce BTK autophosphorylation in matched pre-treatment cells with wild-type BTK from the same patient.

**Figure 6:**
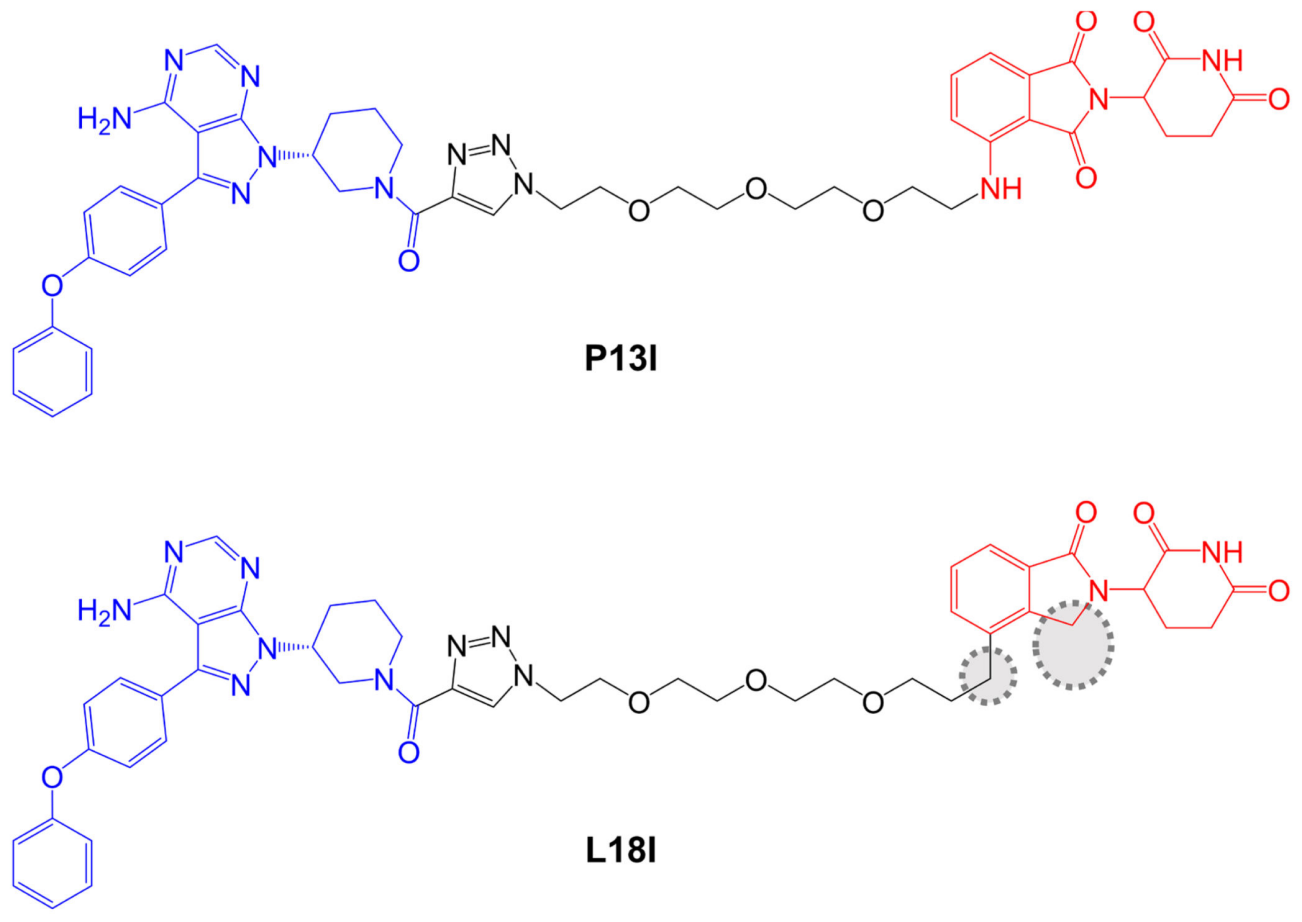
Structures of compounds P13I and L18I. BTK-inhibiting warheads derived from ibrutinib and pomalidomide-derived CRBN-targeting moieties are colored blue and red, respectively [89, 91]. Differences in the structure of L18I compared to P13I are highlighted by dotted regions

Sun et al. [89], described a small series of BTK-targeted PROTACs comprising an ibrutinib-related warhead lacking the acrylamide group or an alternate BTK inhibitor, sperbrutinib [90] with pomalidomide (CRBN-targeted) or RG-71120-derived (MDM2-targeted) E3-binding moieties. Overall, pomalidomide containing PROTACs appeared more promising and further studies focused on P13I which contained the ibrutinib-derived warhead and pomalidomide-derived moieties (Figure 6). P13I effectively reduced BTK expression in various B-cell lines (e.g., DC_50_ ~10 nM after a 3 day incubation in Ramos cells). Neither ibrutinib or pomalidomide induced BTK degradation, whereas ibrutinib, pomalidomide, MG132 or MLN-4924 were able to effectively reduce P13I-induced BTK degradation.

*In vitro* assays demonstrated that P13I inhibited BTK kinase activity with an IC_50_ of ~100 nM and was therefore considerably less effective than ibrutinib (IC_50_ ~0.5 nM). However, P13I was slightly more potent than ibrutinib for growth inhibition of B-cell lymphoma derived HBL1 cells (IC_50_ 1.5 nM for P13I *versus* 2.5 nM for ibrutinib in a 3 day assay). This is consistent with the idea that relatively weak interactions between PROTACs and target proteins can be off-set by stabilizing protein:protein interactions within the TC [7, 92]. Cycloheximide blocking experiments were used to evaluate the stability of BTK in the presence or absence of P13I and demonstrated that P13I accelerated the turn-over of both wild-type and Cys^481^Ser mutant BTK. P13I also inhibited the growth of HBL1 cells with enforced overexpression of Cys^481^Ser mutant BTK, whereas ibrutinib was relatively inactive (IC_50_ ~30 nM for P13I *versus* 700 nM for ibrutinib). Finally, the authors demonstrated that, unlike ibrutinib, P13I did not inhibit *in vitro* activity or induce degradation of ITK or EGFR, known targets of ibrutinib.

**Figure 7:**
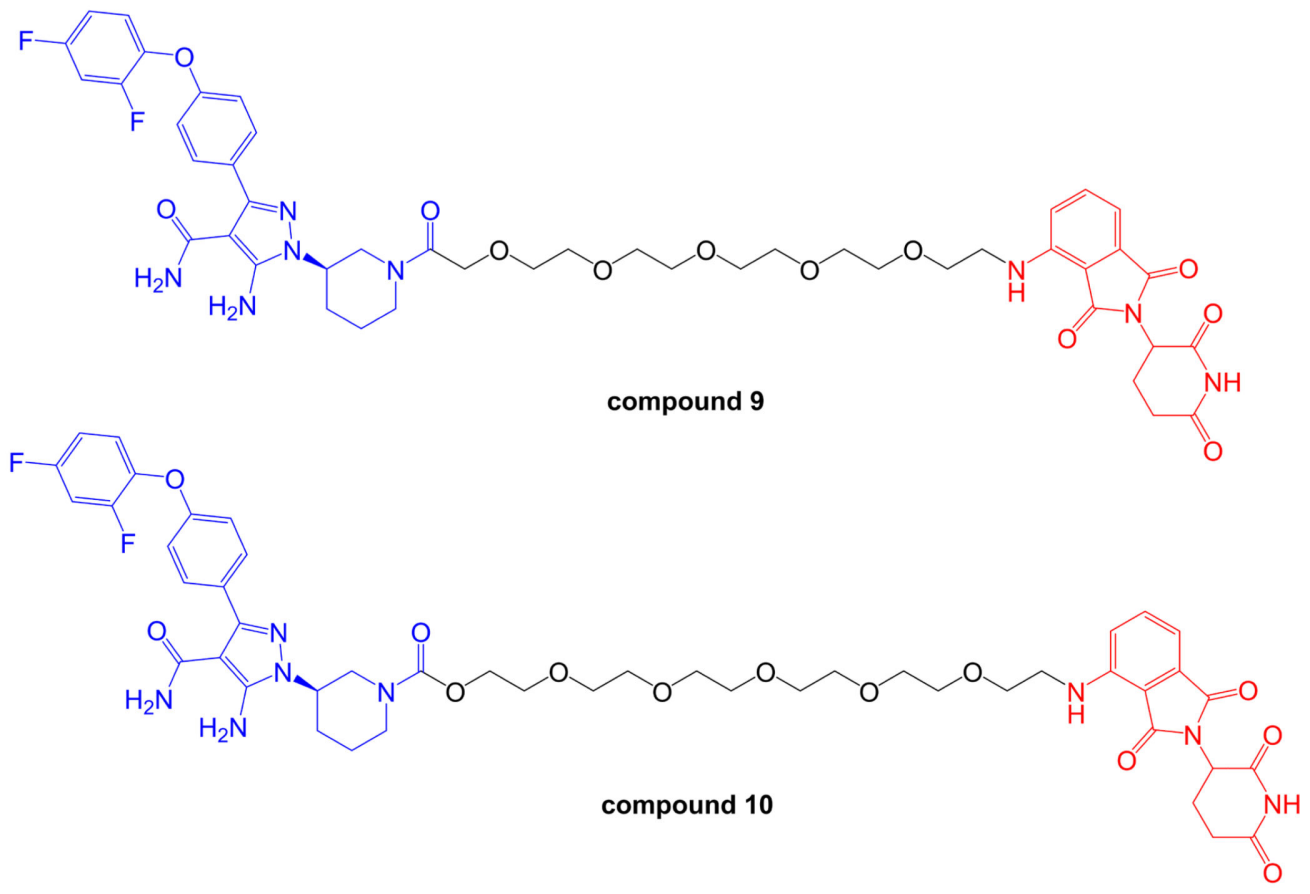
Structures of compounds 9 and 10. BTK-inhibiting warheads are colored blue and pomalidomide-derived CRBN-targeting moieties are colored red [93]. Note that compound 10 differs from compound 9 by having a longer linker

Zorba et al. [93], described a detailed series of experiments to probe the importance of TC formation and inter-element co-operativity in the design of effective BTK-targeted PROTACs. These authors synthesized a small library of compounds comprising a previously disclosed reversible BTK inhibitor [94] and a pomalidomide-derived CRBN-targeting moiety joined by PEG-based linkers of differing lengths (Figure 7). A prototypical compound (compound 9 in that study), with an 18-atom linker substantially reduced BTK expression in Ramos cells (DC_50_ ~6 nM at 24 h) with evidence for a clear hook effect at higher concentrations (> 1 µM). Similar to the study of Buhimschi et al. [87], PROTACs based on recruitment of alternate E3 ligases (VHL and IAP) were much less effective at downregulating BTK expression compared to CRBN-recruiting compounds.

This series of compounds was used to address the role of co-operativity in PROTAC activity. A previous study by Gadd et al. [7], demonstrated the importance of stabilizing interactions between the target and E3 ligase within the TC for effective and selective degradation of the bromo-domain containing protein, BRD4. Comparison of BTK-targeted PROTACs with variable linkers demonstrated that reduction in linker length below 11 atoms strongly ablated BTK degrading activity. Fluorescence resonance energy transfer analysis demonstrated that this loss of BTK degrading activity was tightly correlated with loss of proximity binding of BTK and CRBN. Thus, short linkers appeared to lead to steric clashes which limited TC formation and effective PROTAC activity. Moreover, the authors were unable to demonstrate co-operative BTK-CRBN binding. Thus, at least for this series of compounds, co-operative target-ligase binding does not appear to be required for effective target degradation.

More detailed biological characterization was performed using Ramos cells treated with 1 µM compound 9 or compound 10 for 24 h. Quantitative proteomic analysis demonstrated that these compounds reduced expression of remarkably few proteins in addition to BTK. Other degradation targets identified, including ZFP91, IKZF1 and IKZF3, all of which are transcription factors and were speculated to be natural targets for CRBN-mediated degradation. *In vivo* effects were investigated following sub-cutaneous administration in rats. Interestingly, reduced BTK expression was observed in spleen, but not lung tissues, despite similar accumulation of compound in both sites. The reasons for this observation are not known, but might reflect tissue-specific variation in expression of components of the ubiquitin-proteasome system.

**Figure 8:**
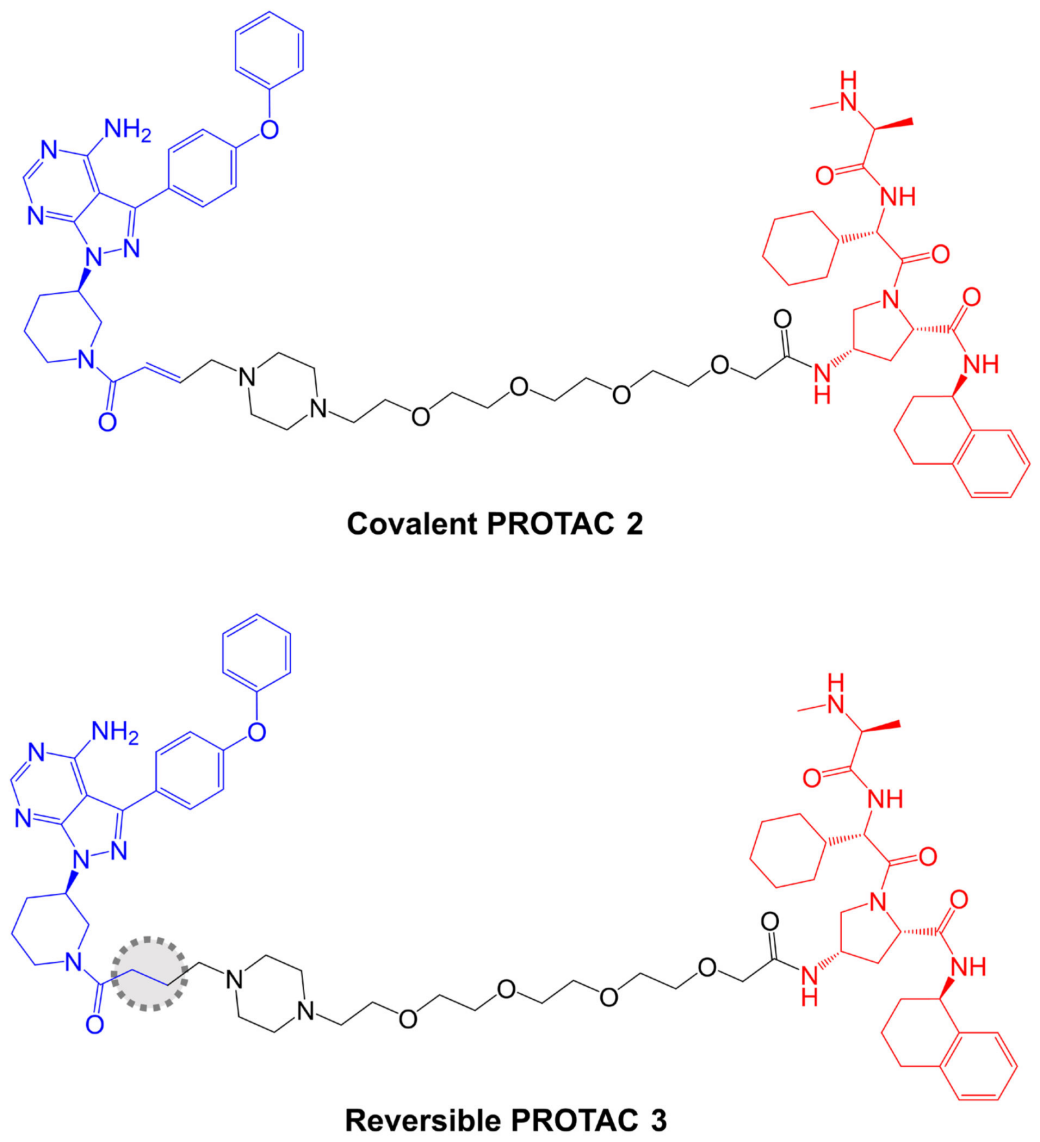
Structures of covalent PROTAC 2 and reversible PROTAC 3. BTK-inhibiting warheads derived from ibrutinib are colored blue and IAP-targeting moieties are colored red [95]. The difference in the structure of the reversible PROTAC 3 compared to covalent PROTAC 2 is highlighted by a dotted region

The potential impact of covalent modification of BTK by a BTK-targeted PROTAC was addressed first by Tinworth et al. [95], by comparing PROTACs containing irreversible and non-covalent ibrutinib-derived warheads and an IAP-recruiting moiety (Figure 8). As expected, both PROTACs inhibited BTK activity in *in vitro* assays and the covalent, but not reversible PROTAC, was demonstrated to conjugate to recombinant BTK using mass spectrometry. Cell-based studies were performed using THP1 cells, derived from AML, and demonstrated that only the non-covalent PROTAC resulted in reduced BTK expression (DC_50_ ~200 nM in a 16 h assay). Thus, despite being able to effectively inhibit BTK activity, the covalent PROTAC was not able to promote BTK degradation. The authors suggested that covalently bound PROTAC may block ubiquitin transfer to the target or access to the proteasome [95].

Further analysis was performed in Ramos cells treated with anti-IgM to stimulate BCR signaling. The covalent PROTAC reduced BTK Tyr^223^ autophosphorylation, consistent with inhibition of BTK kinase activity, but failed to induce BTK degradation. By contrast, the reversible PROTAC relatively weakly reduced BTK Tyr^223^ autophosphorylation (likely due to lower enzyme inhibitory activity associated with reversible binding), but did induce BTK degradation.

Effects of these reversible and covalent BTK-targeted PROTACs on protein degradation were investigated using mass spectrometric analysis of Ramos cells (without anti-IgM stimulation). These experiments were performed using PROTACs with CRBN-recruiting moieties and alternate linker structures. The covalent PROTAC did not affect BTK degradation, but induced degradation of LCK and CSK, known targets of ibrutinib, when tested at 10 µM, suggesting that some targets may be amenable to targeted degradation via covalent PROTACs. The reversible PROTAC also induced degradation of LAT2, CD19 and INPP5D.

**Figure 9:**
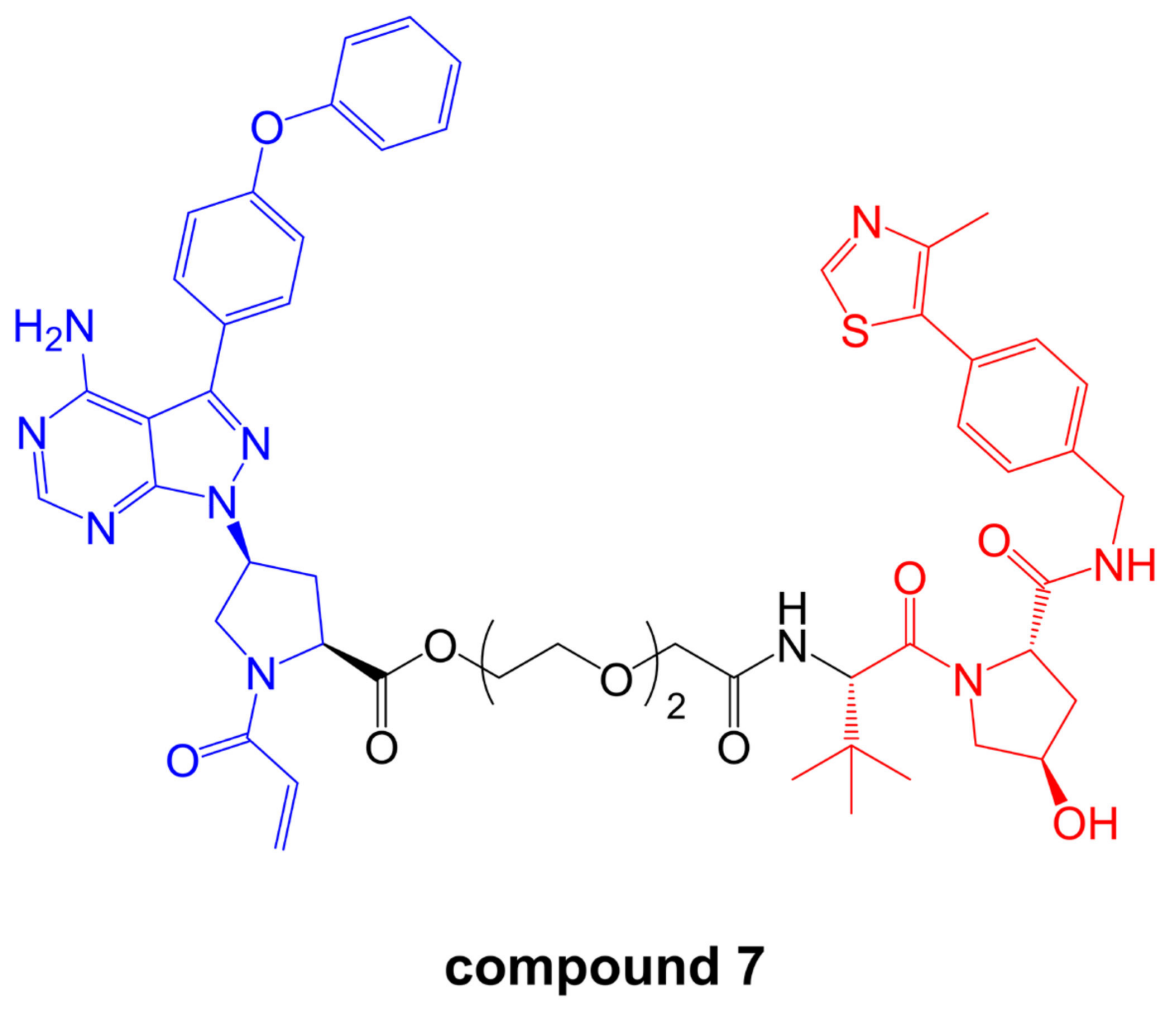
Structure of covalent PROTAC compound 7. BTK-inhibiting warhead related to ibrutinib is colored blue and VHL-targeting moiety is colored red [96]

In contrast to these studies, a more recent report has reported effective BTK degradation by a covalent PROTAC [96]. Compound 7 in the series (Figure 9), comprising an ibrutinib-related BTK inhibitor retaining the acrylamide group [97] linked to a VHL-targeting moiety, induced BTK degradation in K562 cells (derived from chronic myelogenous leukemia) with a DC_50_ of ~150 nM in an 18 h assay.

As discussed above, optimization of physiochemical properties of PROTACs will be critical to move the field from exploration of chemical probes towards clinical exploitation. Studies have demonstrated that, despite their relatively large size and other physiochemical limitations, targeted medicinal chemistry efforts can lead to improved activity for PROTACs [12]. Although this is at a relatively early stage for BTK-targeted PROTACs, important progress has been made in improving pharmacokinetic (PK) properties. A recent follow-on study from the Rao group reported compound L18I which is closely related to P13I (Figure 6) [91]. Similar to P13I, L18I also induced degradation of Cys^481^Ser BTK in HBL1 cells with a DC_50_ of ~30 nM at 36 h. In addition, L18I induced degradation of other BTK variants with Thr, Gly, Trp or Ala substitutions at Cys^481^. L18I was suitable for *in vivo* administration and, in contrast to ibrutinib, significantly reduced the accumulation of Cys^481^Ser BTK-expressing HBL1 cells in a mouse xenograft model with no evidence for substantial toxicities as assessed by body weight. Moreover, L18I was well tolerated (no deaths) in a short term, acute toxicity study in B6 mice.

Jaime-Figueroa et al. [88], also reported a medicinal chemistry effort aimed at improving PK properties of MT-802. Similar to other studies, replacement of the CRBN-targeting moiety with a VHL-targeting ligand resulted in substantially reduced activity. However, replacement of the amide group that connected the linker to the pomalidomide moiety in MT-802 with an ether and removal of one of the carbonyls within the pomalidomide moiety resulted in compound SJF620 (Figure 5) with retained BTK-degrading activity and improved PK properties. For example, following intravenous administration in mice, SJF620 had a half-life of 1.64 h and a C_max_ of 2.1 µg/mL, compared to values of 0.119 h and 0.073 µg/mL for MT-802.

## Conclusion

Consistent with its key role in mediating activation of PLCγ2 and Ca^2+^ mobilization, BTK is an attractive target to interfere with survival and proliferation-promoting signaling downstream of the BCR. Indeed, the irreversible BTK inhibitor ibrutinib has substantially improved outcomes for patients with CLL and some other B-cell malignancies. However, clinical utility of ibrutinib is limited by toxicity and development of resistance, and there is a continuing need for new treatment options. Here, we have reviewed current progress in development of BTK-targeted PROTACs, with a particular focus on their potential to address clinically-relevant drawbacks associated with ibrutinib.

Overall, PROTACs appear to offer an effective, alternate strategy to inhibit BTK activity in CLL. In particular, several agents have been shown to retain activity against Cys^481^Ser mutant BTK, a major cause of clinical ibrutinib resistance [87, 89]. Moreover, BTK-targeted PROTACs appear to be more selective than ibrutinib, even when using very similar (ibrutinib-related) warheads. This has been ascribed to improved selectivity associated with non-covalent binding to BTK and may also reflect stabilizing interactions between BTK and E3 ligase within the TC. Enhanced selectivity has been reported for PROTACs in a number of other studies [2, 6, 8]. Whether this improved selectivity equates to reduced toxicity will require evaluation in appropriate *in vivo* models, particularly to address the most serious ibrutinib-associated toxicities, including atrial fibrillation and bleeding. Although development of PROTACs with suitable PK properties can be challenging, especially for oral administration [98], more recent studies have generated agents suitable for *in vivo* administration with evidence for *in vivo* anti-tumor activity [88, 91, 93].

These studies of BTK-targeted PROTACs have also revealed important features for consideration for the development of effective BTK degrading agents. Two studies have addressed the effectiveness of covalent BTK-targeted PROTACs with differing results [95, 96]. The reasons for these differences are not clear, but there are many differences between the compounds tested and assays used to evaluate activity. Thus, it remains unclear to what extent covalent binding may be generally compatible with BTK inhibition by PROTACs.

As previously discussed [2, 99], studies of BTK-targeted PROTACs confirm the importance of appropriate selection of both linker and E3 ligase recruiting moiety. Overall, CRBN-recruiting moieties seem most effective for BTK targeting [87, 89, 93] and this may relate to the inherent flexibility of CRBN [100]. Linker length is also critical, although the optimal length is dependent on the nature of the coupled warhead and E3-binding domain. This is clearly illustrated in the study by Buhimschi et al. [87], where linker tolerability differed depending on the point of attachment to the pomalidomide moiety. As discussed [93], it is also interesting to note that shorter linkers can be tolerated when attached to alternate BTK-targeted scaffolds, where increased length of the target-binding structure may compensate for shorter linker length [8]. Detailed biochemical studies suggest that, at least in the context of the series studied by Zorba et al. [93], the influence of linker length is primarily to avoid negative, steric clashes between target and E3 ligase. This contrasts markedly with previous studies where stabilizing protein-protein interactions with the TC promoted co-operativity [7, 92].

An interesting feature of these studies is the variable detection of a hook effect which is thought to be due to formation of inactive binary PROTAC:target and PROTAC:E3 ligase complexes at high PROTAC concentrations. For example, this phenomenon, was reported for compound 9 and DD-04-015 [8, 93], but not for MT-802 or reversible PROTAC 3 [87, 95]. The hook effect is expected to be countered by co-operative binding within the TC (thereby favoring TC formation over dimers). Although the studies differ in the concentrations and time points analyzed, it is interesting to note that a clear hook effect was observed for compounds described by Zorba et al. [93], where co-operativity within the TC did not seem to operate.

Finally, an interesting feature revealed by *in vivo* studies is that the PROTAC effectiveness differs between tissue sites. Thus, compound 10 in Zorba et al. [93] was able to degrade BTK in the spleen but not lungs of mice, despite similar drug exposure in both sites. This intriguing observation could be due to tissue-specific differences in the expression of components of the ubiquitin system, such as E3 ligases or de-ubiquitinases. Although not yet explored in the context of BTK-targeted PROTACs, it will be interesting to determine whether such differences can be exploited to avoid toxicities associated with BTK inhibition in normal tissues (for example, by selectively degrading BTK in malignant, but not normal cells). A similar approach has recently been described for PROTACs deployed to degrade the anti-apoptotic BCL-XL protein. Thus, VHL-targeted PROTACs degrade BCL-XL in malignant cells but not thrombocytes due to the lack of VHL in these cells, and thereby avoid platelet toxicity associated with BCL-XL inhibition [101].

Overall, PROTACs appear to be an exciting new approach to target BTK with important distinctions to canonical kinase inhibitors exemplified by ibrutinib. However, development is at a very early stage and considerable progress is required to refine these agents and optimize their drug-like properties before progression to clinical testing.

## Figures and Tables

**Table 1:** Key clinical trials with ibrutinib in CLL

Name	Phase	Population	Response	Ref
N/A^a^	IB/II	R/R^b^	71% ORR^c^ by standard response criteria. Additional 18% partial response with persistent lymphocytosis	[62]
Resonate	III	R/R	Demonstrated that ibrutinib was superior to ofatumumab for response rate, PFS^d^ and OS^e^	[63]
N/A	I/IB	TN^f^ ≥ 65 years	90% achieved objective response or partial response with persistent lymphocytosis	[64]
Resonate-2	III	TN ≥ 65 years	Demonstrated that ibrutinib was superior to chlorambucil for overall response rate, PFS and OS	[65]
Helios	III	R/R	Ibrutinib + bendamustine/rituximab superior to bendamustine/rituximab for PFS and OS	[66, 67]
Alliance A041202	III	TN ≥ 65 years	Ibrutinib superior to bendamustine + rituximab for PFS. No difference between ibrutinib or ibrutinib + rituximab for PFS	[68]
ECOG 1912	III	TN ≤ 70 years	Ibrutinib + rituximab superior to CIT for PFS and OS at interim analysis	[69]
iLLUMINATE	III	TN ≥ 65 years or < 65 years with coexisting conditions	Ibrutinib + obinutuzumab superior to chlorambucil + obinutuzumab for PFS	[70]

a N/A: not applicable;

b R/R: relapsed/refractory;

c ORR: overall response rate;

d PFS: progression-free survival;

e OS: overall survival;

f TN: treatment naïve

## Abbreviations

AML: acute myeloid leukemia
BCR: B-cell receptor
BTK: Bruton tyrosine kinase
CaM: calmodulin
CIT: chemoimmunotherapy
CLL: chronic lymphocytic leukemia
CRBN: cereblon
DAG: diacylglycerol
E1: ubiquitin-activating enzyme
E2: ubiquitin-conjugating enzyme
E3: ubiquitin protein ligase
iCa^2+^: intracellular Ca^2+^
IP_3_: inositol 1,4,5-trisphosphate
M-CLL: mutated CLL
NF-κB: nuclear factor-κB
NFAT: nuclear factor of activated T-cells
ORR: overall response rate
OS: overall survival
PD: pharmacodynamic
PFS: progression-free survival
PI(3,4,5)P3: phosphatidylinositol 3,4,5-trisphosphate
PI(4,5)P2: phosphatidylinositol 4,5-bisphosphate
PK: pharmacokinetic
PLCγ2: phospholipase C
PROTAC: proteolysis targeting chimera
R/R: relapsed/refractory
SHM: somatic hypermutation
SLL: small lymphocytic lymphoma
TC: ternary complex
TN: treatment naïe
U-CLL: unmutated CLL
VHL: Von Hippel-Landau
XLA: X-linked agammaglobulinemia
